# Whole Genome Sequence of the *Treponema* Fribourg-Blanc: Unspecified Simian Isolate Is Highly Similar to the Yaws Subspecies

**DOI:** 10.1371/journal.pntd.0002172

**Published:** 2013-04-18

**Authors:** Marie Zobaníková, Michal Strouhal, Lenka Mikalová, Darina Čejková, Lenka Ambrožová, Petra Pospíšilová, Lucinda L. Fulton, Lei Chen, Erica Sodergren, George M. Weinstock, David Šmajs

**Affiliations:** 1 Department of Biology, Faculty of Medicine, Masaryk University, Brno, Czech Republic; 2 The Genome Institute, Department of Genetics, Washington University School of Medicine, St. Louis, Missouri, United States of America; Institut Pasteur, France

## Abstract

**Background:**

Unclassified simian strain *Treponema* Fribourg-Blanc was isolated in 1966 from baboons (*Papio cynocephalus*) in West Africa. This strain was morphologically indistinguishable from *T. pallidum* ssp. *pallidum* or ssp. *pertenue* strains, and it was shown to cause human infections.

**Methodology/Principal Findings:**

To precisely define genetic differences between *Treponema* Fribourg-Blanc (unclassified simian isolate, FB) and *T. pallidum* ssp. *pertenue* strains (TPE), a high quality sequence of the whole Fribourg-Blanc genome was determined with 454-pyrosequencing and Illumina sequencing platforms. Combined average coverage of both methods was greater than 500×. Restriction target sites (n = 1,773), identified *in silico*, of selected restriction enzymes within the Fribourg-Blanc genome were verified experimentally and no discrepancies were found. When compared to the other three sequenced TPE genomes (Samoa D, CDC-2, Gauthier), no major genome rearrangements were found. The Fribourg-Blanc genome clustered with other TPE strains (especially with the TPE CDC-2 strain), while *T. pallidum* ssp. *pallidum* strains clustered separately as well as the genome of *T. paraluiscuniculi* strain Cuniculi A. Within coding regions, 6 deletions, 5 insertions and 117 substitutions differentiated Fribourg-Blanc from other TPE genomes.

**Conclusions/Significance:**

The Fribourg-Blanc genome showed similar genetic characteristics as other TPE strains. Therefore, we propose to rename the unclassified simian isolate to *Treponema pallidum* ssp. *pertenue* strain Fribourg-Blanc. Since the Fribourg-Blanc strain was shown to cause experimental infection in human hosts, non-human primates could serve as possible reservoirs of TPE strains. This could considerably complicate recent efforts to eradicate yaws. Genetic differences specific for Fribourg-Blanc could then contribute for identification of cases of animal-derived yaws infections.

## Author Summary

A bacterial strain isolated in 1966 from baboons (*Papio cynocephalus*) in West Africa was preliminarily characterized as unclassified simian strain *Treponema* Fribourg-Blanc (FB). This strain was morphologically identical to *T. pallidum* ssp. *pallidum* (TPA, agent of syphilis) or ssp. *pertenue* (TPE, agent of yaws). In this study, we completed a high quality whole genome sequence of simian isolate *Treponema* Fribourg-Blanc and compared it to known genome sequences of *Treponema pallidum* strains. No major differences in the gene order of the FB genome were found when compared to all known genomes of *Treponema pallidum* subspecies. Moreover, the FB genome clustered with other TPE strains, while *T. pallidum* ssp. *pallidum* strains clustered separately. In general, the FB genome showed similar genetic characteristics to other TPE strains. Therefore, we proposed that the simian isolate Fribourg-Blanc be classified as a bacterial strain belonging to *Treponema pallidum* ssp. *pertenue*. It appears that, except for humans, the reservoir of yaws-causing treponemes may also include free-living primates, especially in Africa.

## Introduction

*Treponema* Fribourg-Blanc was isolated in 1966 from baboons (*Papio cynocephalus*) in West Africa [1], [2]. This strain was morphologically indistinguishable from *T. pallidum* ssp. *pallidum* (TPA) or ssp. *pertenue* (TPE) strains and the ability to cause human infection was experimentally verified [3]. In baboons, enlarged lymphatic nodes with no specific clinical signs were observed [2]. Several other cases of primate treponematoses have been described [4]–[9] either without clinical signs or with symptoms of yaws. Skin samples taken from baboons in the Gombe National Park revealed a yaws-like infection that appeared to be transmitted via sexual contact [10]. Furthermore, in a field survey in 2007 at Lake Manyara National Park in Tanzania, several olive baboons (*Papio hamadryas anubis*) showed severe ulcerations strictly localized to the anogenital regions [11]. Similar lesions were found also in wild baboons living in other Tanzanian National Parks and in the Ngorongoro Conservation Area (Tanzania) [12]. Although this clinical manifestation suggested a disease similar to human syphilis infections, a genetic analysis of the causative agent showed higher genetic similarity to human yaws-causing strains than to syphilis-causing strains [11], [12].

The causative agent of yaws, *Treponema pallidum* ssp. *pertenue* [13], predominantly causes infections in tropical regions of Africa, Asia, Oceania and South America with an estimated prevalence of 2 million cases worldwide [14]. Three TPE strains were recently sequenced [15] and the observed genetic difference from syphilis-causing strains of *T. pallidum* ssp. *pallidum* was lower than 0.2% of the genome sequence. In humans, yaws is a multi-stage disease, transmitted through direct skin contact from an infected patient to a recipient. It is characterized by skin nodules and ulcerations, joint and soft tissue destruction and bone changes. Although some reports have described infection of the central nervous system, cardiovascular system and fetus during yaws infection [16], there is not enough experimental data to clearly prove the ability of TPE strains to invade the CNS or cause congenital infection. It is generally believed that humans are the primary reservoir of yaws. Since transmission requires direct contact with the causative agent of yaws, a risk of contamination between human and other primates could exist in regions where yaws and other primate treponemal infections occur simultaneously [10].

Several previous genetic studies have described partial FB sequences [17]–[26] and some of them predicted that FB strains were closely related to TPE strains [18], [19], [21], [22], [24]. Prior to this work, about 55 kbp (4.83%) of the FB genome sequence had been determined. In this communication, we compare the complete genome sequence of the simian isolate Fribourg-Blanc to three TPE strains (Samoa D, CDC-2, and Gauthier) and to five TPA strains (Nichols, DAL-1, Chicago, SS14, Mexico A). Based on the low genetic variability between Fribourg-Blanc and the 3 TPE strains, the Fribourg-Blanc bacterial strain is a *Treponema pallidum* ssp. *pertenue* strain.

## Materials and Methods

### Amplification and isolation of Fribourg-Blanc DNA

A sample containing extracted Fribourg-Blanc treponemes (from infected rabbit tissue) was obtained from D. L. Cox, CDC, Atlanta, GA, USA. The sample contained 5×10^6^ cells per ml and the DNA was amplified in one step directly from frozen cells (5×10^3^ cells) with the whole genome amplification procedure (REPLI-g kit, QIAGEN, Valencia, CA, USA). Amplification resulted in 413 ng of DNA per µl, (30 µl in total); however, both treponemal and rabbit DNA was present in the amplified DNA. Therefore, Fribourg-Blanc DNA was repeatedly amplified using the pooled segment genome sequencing (PSGS) method described previously [15]. Briefly, the genomic Fribourg-Blanc DNA was amplified with 134 specific primer pairs as overlapping PCR products (Table S1). To enable sequencing of paralogous genes, PCR products were separated into four pools (pool 1–4) and mixed in equimolar amounts. For 454-pyrosequencing, PCR products of these pools were labeled with multiplex identifier (MID) adapters and sequenced as four different samples. However, only one sequencing mixture was prepared for Illumina because MID adapters were not available.

### DNA sequencing and assembly of the Fribourg-Blanc genome

Whole genome DNA sequencing used a Roche/Genome Sequencer FLX System platform (454 Life Sciences, Branford, CT, USA) combined with the Illumina/Solexa Genome Analyzer IIx approach (Illumina, San Diego, CA, USA). Sequencing was performed at The Genome Institute, Washington University School of Medicine (St. Louis, MO, USA). 454 reads were assembled using a Newbler assembler while Illumina reads were assembled using Velvet [27]. 454-pyrosequencing and Illumina sequencing resulted in average read lengths of 230 bp and 35 bp and the total average coverage of 70× and 465×, respectively. Assembled contigs obtained from both methods were aligned to the reference genome TPE CDC-2 using Lasergene software (DNASTAR, Madison, WI, USA). All gaps in the genome sequence and all discrepancies between contig sequences obtained using both methods were resolved using Sanger sequencing. Altogether, 85 genomic regions of the Fribourg-Blanc genome were amplified and Sanger sequenced.

In addition, several genomic regions were amplified with specific primers as *Treponema pallidum* intervals (TPI) using a GeneAmp XL PCR Kit (Applied Biosystems, Foster City, CA, USA) [28]. These intervals contained following paralogous genes: *tprC* (TPI11), *tprD* (TPI12), *tprE* (TPI25A), *tprF* and *tprG* (TPI25B), *tprI* and *tprJ* (TPI48), and *tprL* (TPI78). XL PCR products were purified using a QIAquick PCR Purification Kit (QIAGEN) and sequenced with a BigDye Terminator v3.1 Cycle Sequencing Kit (Applied Biosystems) using internal primers. The TPI71A (Table S1) region, which was not included in any pool, was sequenced similarly. The *tprK* (TPFB_0897), *arp* (TPFB_0433), and TPFB_0470 genes were amplified and cloned into pCR 2.1-TOPO (Invitrogen, Carlsbad, CA, USA) and five independent clones for TPFB_0433, eight for TPFB_0470 or ten clones for *tprK* were sequenced.

A total of 11 genomic regions (in genes TPFB_0012, TPFB_0040, TPFB_0067, TPFB_0179, TPFB_0279, TPFB_0347, TPFB_0859, TPFB_0865 and in intergenic regions (IGR) TPFB_0347–0348, IGR TPFB_0379–0380, IGR TPFB_0381–0382), containing homopolymeric (G or C) stretches were amplified with *Pfu* polymerase (Fermentas Inc., Glen Burnie, MD, USA) as follows: 5 µl of 10× *Pfu* buffer with 20 mM MgSO_4_, 1 µl of dNTP mix (each nucleotide of 10 mM concentration), 1 µl of DNA (1–5 ng/µl), 0.5 µl of forward primer and 0.5 µl of reverse primer, 41 µl of water for PCR, and 1 µl (2.5 U) of *Pfu* DNA polymerase. The cycling conditions were: 94°C for 1 minute; 30 cycles: 94°C for 1 minute, 60°C for 30 s, 72°C for 1 minute; 72°C for 10 minutes. To facilitate the subsequent cloning of these PCR products into a pCR 2.1-TOPO vector (Invitrogen), 0.2 µl of *Taq* polymerase was added to the mixture and incubation at 72°C for 10 minutes followed. Plasmid DNA was isolated using a QIAGEN Plasmid Mini Kit (QIAGEN) and sequenced with universal primers from a TOPO TA Cloning Kit. At least five independent clones were sequenced.

### Whole genome fingerprinting (WGF)

To verify final genome assemblies, whole genome fingerprints of three enzymes including *Bam*H I, *Eco*R I and *Hin*d III [24], [28] were compared to the *in silico* restriction enzyme analysis of the sequenced Fribourg-Blanc genome. The average error rate of WGF for *Treponema paraluiscuniculi* strain Cuniculi A was previously calculated [29] and corresponded to 27.9 bp (1.6% of the average fragment length) with a variation range between 0 and 132 bp.

### Gene identification, annotation and classification

The final whole genome sequence was assembled from 454-pyrosequencing and Illumina contigs and Sanger sequenced regions comprising the *tpr* genes, repetitive DNA regions (e.g. TPFB_0433, TPFB_0470), regions containing homopolymers, gaps between contigs and discrepant regions between 454 and Illumina contigs. The Geneious software v5.6.5 [30] was used for gene annotation based on the recent annotation of the CDC-2 genome [15]. Gene TPFB_0897, coding for TprK protein, showed intrastrain variability and therefore nucleotides in variable regions were replaced with Ns in the complete genome sequence. Genes were tagged with the TPFB_ prefix. In Fribourg-Blanc, the original locus tag numbering corresponds to the tag numbering of orthologous genes annotated in the TPE CDC-2 genome. For proteins with unpredicted functions, a gene size limit of 150 bp was applied. TPE genes were classified into seven groups according to their probable function as described previously, i.e. genes involved in general metabolism; in cell processes and cell structure; in DNA replication, repair, recombination; in regulation, transcription and translation; in transport; in virulence; and genes of unknown function [15].

### Comparisons of whole genome sequences

Whole genome nucleotide alignments of five TPA strains, three TPE strains [15], *Treponema paraluiscuniculi* Cuniculi A strain (CP002103.1, [29]) and the Fribourg-Blanc isolate (CP003902.1) were used for determination of genetic relatedness using several approaches including calculation of nucleotide diversity (π), calculation of nucleotide divergence (d_A_) and construction of a phylogenetic tree. TPA strains included Nichols (resequenced genome; unpublished data), DAL-1 (CP03115.1, [31]), SS14 (resequenced genome; unpublished data), Chicago (CP001752.1, [32]), and Mexico A (CP003064.1, [33]) while TPE strains included Samoa D (CP002374.1), CDC-2 (CP002375.1), and Gauthier (CP002376.1). Whole genome alignments were constructed using Geneious software and SeqMan software (DNASTAR, Madison, WI, USA). Nucleotide changes among studied whole genome sequences were analyzed using *DnaSP* software, version 5.10 [34]. An unrooted phylogenetic tree was constructed from whole genome sequence alignments using the Maximum Parsimony method and MEGA software [35].

### Nucleotide sequence accession numbers

The complete genome sequence of the Fribourg-Blanc isolate was deposited in the GenBank under the accession number CP003902.1.

## Results

### Whole genome sequencing, genome annotation, and genomic parameters

The FB genome was determined using two independent whole genome sequencing methods (454-pyrosequencing, Illumina) with a total combined average coverage greater than 500×. The Sanger sequencing method was used for finishing the complete genome sequence and for additional sequencing including paralogous, repetitive and intrastrain variable chromosomal regions.

The Fribourg-Blanc genome was annotated according to the sequence of the CDC-2 genome [15]. The gene names were denoted with the TPFB_ prefix (*Treponema pallidum* Fribourg-Blanc). The FB genome was most similar to TPE strains. The summarized genomic features of the Fribourg-Blanc simian isolate (and other completely sequenced TPE strains) are shown in Table 1. The Fribourg-Blanc genome (1,140,481 bp) was 737–1151 bp longer than other TPE strains. No major genome rearrangements were found compared to the other 3 TPE genomes. Altogether, 1122 genes were annotated in the Fribourg-Blanc genome including 54 untranslated genes encoding rRNA, tRNA and other ncRNA (a short bacterial RNA molecules that are not translated into a protein). Compared to the other TPE genomes, genes TPFB_0012 and TPFB_0896 (both encoding hypothetical proteins) contained a 1 bp deletion (frameshift mutation) and nonsense mutation, respectively. Therefore, these two genes were not annotated in the FB genome. TPFB_0304 (encoding treponemal conserved hypothetical protein) was not annotated because of nucleotide change in the stop codon followed by fusion with the TPFB_0303 (encoding DNA mismatch repair protein MutL). The average and median gene lengths of the Fribourg-Blanc genome were calculated as 983 bp and 831 bp, respectively. The intergenic regions covered 53 kb and represented 4.63% of total FB genome length, which is similar to the length of these regions in other TPE strains. A total of 640 genes (57.0%) encoded proteins with predicted function, 139 genes encoded treponemal conserved hypothetical proteins (TCHP, 12.4%), 141 genes encoded conserved hypothetical proteins (CHP, 12.6%), 145 genes encoded hypothetical proteins (HP, 12.9%) and 3 genes (0.3%) were annotated as pseudogenes. When compared to the Nichols genome (AE000520.1), 9 additional genes (orthologous to TP0129, TP0132, TP0180, TP0266, TP0318, TP0370, TP0532, TP0671 and TP1030) can be considered as pseudogenes in the Fribourg-Blanc genome (the same genes were also considered pseudogenes in other TPE strains). When compared to TPE strains, 2 additional genes (orthologous to TPE_0012, TPE_0896; Table 2) can be considered pseudogenes in the FB genome.

**Table 1:** Summary of genomic features of the FB genome and three *T. pallidum* ssp. *pertenue* strains (Samoa D, CDC-2 and Gauthier).

Genome parameter	Fribourg-Blanc isolate	Samoa D	CDC-2	Gauthier
GeneBank accession number	CP003902.1	CP002374.1	CP002375.1	CP002376.1
Genome size	1,140,481 bp	1,139,330 bp	1,139,744 bp	1,139,417 bp
G+C content	52.80%	52.80%	52.80%	52.80%
No. of fused genes^a^	25 (52 corresponding genes in the Nichols genome)	25 (52 corresponding genes in the Nichols genome)	24 (50 corresponding genes in the Nichols genome)	24 (50 corresponding genes in the Nichols genome)
Sum of the intergenic region lengths (% of the genome length)	52,785 bp (4.63 %)	52,844 bp (4.64%)	52,963 bp (4.65%)	53,300 bp (4.68%)
Average/median gene length	982.6/831.0 bp	980.3/831.0 bp	980.4/831.0 bp	979.3/831.0 bp
No. of predicted protein-encoding genes	1065	1068	1068	1068
No. of genes encoded on plus/minus DNA strand	599/523	600/525	600/525	600/525
No. of genes coding for proteins with predicted function	640	640	640	640
No. of genes coding for treponemal conserved hypothetical proteins	139	140	140	140
No. of genes coding for conserved hypothetical proteins	141	141	141	141
No. of genes coding for hypothetical proteins	145	147	147	147
No. of annotated pseudogenes (no. of all pseudogenes compared to Nichols sequence^a^)	3 (14)	3 (12)	3 (12)	3 (12)
No. of tRNA loci	45	45	45	45
No. of rRNA loci	6 (2 operons)	6 (2 operons)	6 (2 operons)	6 (2 operons)
No. of ncRNAs	3	3	3	3

a Number of genes in a particular genome which sequence include at least 2 genes predicted in the Nichols genome AE000520.1.

**Table 2:** Mutations causing gene changes resulting in protein truncations and elongations in comparison with TPE strains.

Gene (predicted protein function)	Nucleotide change	Coordinates of change in the FB genome (CP003902.1)	Result of nucleotide change
TPFB_0012 (HP)	1 bp deletion^a^	12479–12487	gene shortened by 47 bp to 129 bp, gene was not annotated in the FB genome
TPFB_0040, *mcp* (methyl-accepting chemotaxis protein)	5 bp insertion^a^	49359–49373	gene shortened by 17 bp to 2433 bp
TPFB_0126b (HP)	3 bp substitution in the start codon	148982–148984	gene shortened by 42 bp to 366 bp
TPFB_0303 (TCHP)	1 bp substitution in the stop codon	319012	gene fusion of genes ortologous to TPE_0303 and TPE_0304, gene was annotated as TPFB_0303 (5076 bp)
TPFB_0347 (HMP)	2 bp insertion^a^	373747–373761	gene shortened by 35 bp to 711 bp
TPFB_0433, *arp* (Arp protein)	15 tandem repeat units, one unit was 60 bp long	462777–463676	Samoa D, CDC-2, and Gauthier, contains 12, 4, and 10 repeat units, respectively
TPFB_0461a (HP)	1 bp deletion^a^	493013–493022	gene elogation by 61 bp to 243 bp
TPFB_0470 (CHP)	22 tandem repeat units, one unit 24 bp long	499435–499962	Samoa D, CDC-2, and Gauthier, contains 12, 37, and 25 repeat units, respectively
TPFB_0484 (CHP)	1 bp deletion^a^	517701–517708	gene shortened by 309 bp to 1707 bp
TPFB_0548 (TCHP)	42 bp deletion	594092–594093	gene shortened by 42 bp to 1257 bp
TPFB_0896 (HP)	2 bp substitution leading to nonsense mutation	977039, 977041	gene shortened by 99 bp to 54 bp, gene was not annotated in the FB genome

HP- hypothetical protein, CHP – conserved hypothetical protein, TCHP – treponemal conserved hypothetical protein, HMP – hypothetical membrane protein.

a changes in homopolymeric regions.

### Whole genome fingerprinting

The *in silico* identified restriction target sites (RTS) within the FB genome were compared with experimental restriction digest patterns of individual TPI regions covering the entire TPE genome [24]. Altogether, 1,773 RTSs representing more than 10.6 kb of analyzed sequence were experimentally tested [24]. Since no discrepancies between *in silico* and experimental RTS analyses of the FB genome were found, the estimated sequencing error rate for the FB genome was therefore 10^−4^ or less.

### Sequence relatedness of the FB genome to other pathogenic treponemal genomes

**Figure 1:**
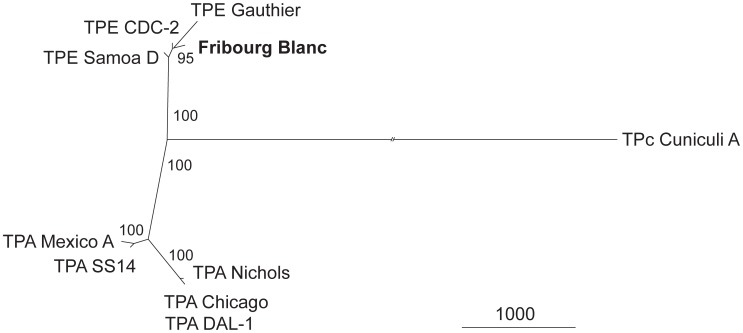
An unrooted tree constructed from whole genome sequence alignments of 10 complete genome nucleotide sequences. An unrooted tree constructed from whole genome sequence alignments using the Maximum Parsimony method and MEGA software [34]. The bar scale corresponds to 1000 nt changes. Bootstrap values based on 1,000 replications are shown next to the branches. All positions containing deletions in at least one genome sequence were omitted from further analysis. The analysis comprised 10 complete genome nucleotide sequences including 5 strains of TPA (*Treponema pallidum* ssp. *pallidum*), 3 strains of TPE (*Treponema pallidum* ssp. *pertenue*), one TPc (*Treponema paraluiscuniculi*) strain and the FB strain. There were a total of 1,129,016 nucleotide positions aligned in the final dataset. Note the clustering of the FB genome with other TPE strains. The branch of TPc was shortened (//).

Sequence relatedness of the FB genome to other TP genomes based on available whole genome sequences is shown in Figure 1. The FB genome clustered with other TPE strains (especially with the TPE CDC-2 strain), while TPA strains, as well as the genome of the *T. paraluiscuniculi* (TPc) strain Cuniculi A, each clustered separately. Calculated nucleotide diversity among currently sequenced *T. pallidum* and *T. paraluiscuniculi* strains are shown in Table 3. Detailed characterization of nucleotide diversity between TPE strains and the FB isolate is shown in Table 4. The FB genome was found to be 99.97% identical to other TPE genomes. The lowest calculated nucleotide diversity (π) ± standard deviation among TPE strains and the FB isolate was found between the Fribourg-Blanc and CDC-2 strain (0.00016±0.00008), which is identical to nucleotide diversity between Samoa D and CDC-2 genomes. In contrast, the highest calculated nucleotide diversity was found between the Fribourg-Blanc and Gauthier strain (0.00044±0.00022), which was similar to the difference between the Samoa D and and Gauthier strains (0.00044±0.00022). For comparison, calculated π values between Fribourg-Blanc and TPA strains were one order of magnitude higher than π values between Fribourg-Blanc and TPE strains (Table 3).

**Table 3:** Calculated nucleotide diversity (π± standard deviation) between FB isolate and individual TPA strains, TPE strains and the Cuniculi A strain.

Strain	Nucleotide diversity
TPA Nichols	0.00206±0.00103
TPA DAL-1	0.00209±0.00104
TPA Chicago	0.00203±0.00102
TPA SS14	0.00180±0.00090
TPA Mexico A	0.00172±0.00086
TPE Samoa D	0.00023±0.00012
TPE CDC-2	0.00016±0.00008
TPE Gauthier	0.00044±0.00022
TPc Cuniculi A	0.01044±0.00518

**Table 4:** Calculated nucleotide diversity (π± standard deviation) between individual TPE strains and the FB isolate.

Fribourg-Blanc	Gauthier	CDC-2	Samoa D	
0.00023±0.00012	0.00044±0.00022	0.00016±0.00008	***	Samoa D
0.00016±0.00008	0.00037±0.00018	***		CDC-2
0.00044±0.00022	***			Gauthier
***				Fribourg-Blanc

### Genome differences specific for the FB genome

To define genome differences specific to the FB genome, the whole genome sequence of this strain was compared to the available genome sequences of TPE strains [15]. In coding regions, 6 deletions, 5 insertions and 117 substitutions differentiated FB from TPE genomes (Table 5, Table S2). Frameshift mutations (three deletions and two insertions) resulted in an ommitted annotation of TPFB_0012 (encoding hypothetical protein), in gene truncation (TPFB_0040, *mcp* coding for methyl-accepted chemotaxis protein; TPFB_0347 encoding hypothetical membrane protein; TPFB_0484, encoding conserved hypothetical protein) or in gene elongation (TPFB_0461a, encoding hypothetical protein). Other major changes were located in genes TPFB_0548 (containing 42-bp deletion), TPFB_0303 fused with TPFB_0304, TPFB_0126b (truncated as a result of a start codon mutation), TPFB_0896 (not annotated because of a nonsense mutation), TPFB_0433 encoding acidic repeat protein Arp (containing 15 tandem repeat units of 60-bp compared to 12, 4, and 10 repeat units in Samoa D, CDC-2, and Gauthier, respectively) and TPFB_0470 (containing 22 tandem repeat units of 24-bp, compared to 12, 37, and 25 tandem repeat units in Samoa D, CDC-2, and Gauthier, respectively). A set of 117 substitutions resulted in one nonsense mutation, one mutation affecting the start codon, one mutation affecting the stop codon and in 88 nonsynonymous mutations (82 nonconserved). Most of the changes were found in *tprC* (TPFB_0117) and *tprI* (TPFB_0620) genes. Mutations causing changes larger than 5 amino acid replacements or protein truncations and elongations are listed in Table 2.

**Table 5:** Genome differences specific for the FB genome (comprising 630 nucleotides).

	Non-coding sequences (IGR) (altogether 445 bp)		Coding sequences (altogether 185 bp)		
Nucleotide difference	Number of changes	Number of mutated nucleotides	Number of changes	Number of mutated nucleotides (relevant protein change)	The most affected gene(s)
deletion	3	2× single bp,1×2 bp, altogether 4 bp	6	3× single bp, altogether 3 bp (protein truncation or elongation); 3, 6 and 42 bp, altogether 51 bp; (protein shortening)	TPFB_0012, 0461a, 0484; TPFB_0370, 0548, 0859
insertion	3	2×2 bp, 1×430 bp (TPFB_0696–TPFB_0697), altogether 434 bp	5	1×2 bp, 1×5 bp, altogether 7 bp (protein truncation); 3×3 bp, altogether 9 bp; (protein elongation)	TPFB_0347, 0040; TPFB_0179, 0279, 0462
substitution	7	7× single bp, altogether 7 bp	117	117 bp (synonymous or nonsynonymous mutations)	TPFB_0117, 0126a, 0316, 0324, 0488, 0620, 0865, 0968

Genome differences specific for the FB genome (comprising 630 nucleotides) in both non-coding (intergenic regions, IGR) and coding regions when compared to TPE strains (Samoa D, CDC-2, Gauthier).

The *tprK* gene was excluded from this list of changes because of high sequence diversity within TPE strains.

## Discussion

The complete genome sequence of the simian isolate Fribourg-Blanc (FB) was determined and compared to five syphilis-causing (TPA) and three human yaws-causing *T. pallidum* ssp. *pertenue* (TPE) strains. Previous reports have shown that the FB strain (isolated from *Papio cynocephalus* in 1966 in West Africa) was morphologically indistinguishable from other TPA or TPE strains [2]. Moreover, the ability of FB strain to attach to mammalian cells was similar to TPE but different from TPA strains [36]. In addition to these studies, several other genetic studies showed a close relationship between FB strain and TPE strains of human origin [18], [19], [21], [22], [24].

Experimental human and monkey (monkeys of the genus *Macaccus*) infection with the FB strain resulted in symptoms similar to yaws [3], [37]. Conversely, primate infection with TPE strains of human origin resulted in detectable lesions in at least a subset of infected monkeys of the genus *Macacus* and *Semnopithecus* [38]. In addition, several other reports demonstrated that TPE strains of human origin can experimentally infect monkeys [39], [40]. These data indicate that both TPE and FB strains have overlapping or identical host range suggesting a close relationship among these strains.

As shown by this study, the genome of the FB simian isolate [1], [2] was very similar to TPE strains of human origin. Although the FB genome was most closely related to the African TPE CDC-2 strain (isolated in Akorabo, Ghana in 1980 [41]), its relatedness to another TPE strain of African origin, strain Gauthier (isolated in Brazzaville, Congo in 1960 [42]) was lower compared to the TPE Samoa D strain (isolated in Western Samoa in 1953 [43]). Thus, the Gauthier strain was the most distinct among the TPE strains. Compared to TPA strains (Nichols, SS14, DAL-1, Chicago, Mexico A), the calculated nucleotide diversity between individual TPE strains and the FB isolate was one order of magnitude lower than between TPA strains and the FB isolate. These data suggest that the FB isolate is in fact another TPE strain.

Several previous studies described partial FB sequences [17]–[26]. Altogether, 38 Fribourg-Blanc sequences comprising 55066 bp (4.83% of the FB genome sequence) were found when searching databases. Altogether, 7 nucleotide discrepancies in our genomic sequence were identified, and most of them were located in *tpr* genes or their vicinity (n = 4) and in homopolymeric regions (n = 2). Analysis of individual sequencing reads in these regions in Illumina, 454-pyrosequencing and Sanger raw data (except for homopolymeric regions where 454 pyrosequencing reads were not considered relevant) supported the sequences presented by our research. Besides differences in *tpr* regions and in homopolymeric regions that are likely results of intrastrain heterogeneity [31], [44]–[46], a single remaining difference was found in the gene TPFB_1038 (*tpF-1*, GenBank acc. no. EU102242, [22]). This difference may represent a genetic difference between different passages of the FB strain, locus with intrastrain heterogeneity or sequencing error.

Specific changes (deletions, insertions, and substitutions) comprising 185 nucleotides in 68 genes differentiated the FB strain from other TPE strains. Major genetic changes between FB and TPE genomes resulting in protein truncations or elongations were located in 9 genes. These genes encoded hypothetical proteins with the exception of TPFB_0040 (encoding methyl-accepting chemotaxis protein, Mcp). Moreover, the genome of the FB strain contained a different number of tandem repeat units in genes TPFB_0433 (encoding the acidic repeat protein, Arp) and TPFB_0470 (encoding a conserved hypothetical protein) compared to orthologous genes in individual TPE strains. The number and sequence of 60-bp tandem repeat units within the *arp* gene, in the FB genome, revealed the same pattern as previously described for this strain [23]. Variability in the number of tandem repeat units in genes orthologous to TPFB_0470 was also described in TPE and TPA strains [15], [24]. A relatively high expression rate of the TP0470 gene, a TPFB_0470 ortholog, in the Nichols genome during experimental rabbit infection was found [47]. This fact together with the variable number of tandem repeat units in this gene indicates that this gene may be involved in pathogen-host interactions. In bacterial pathogens, highly synthesized proteins with a variable number of tandem repeats are often involved in interaction with the host, e.g. an abundant outer membrane protein, secretin PilQ, of *Neisseria meningitidis* contains four to seven octapeptide copies and is a potential vaccine candidate for serogroup B of *N. meningitidis* [48]. In all genes with affected length in the FB genome (except for TPFB_0484 and TPFB_0347), major sequence differences were also found in the orthologous genes in TPE, TPA, and Cuniculi A genomes [29]. Moreover, the TPFB_0304 gene was also found variable among orthologous genes in TPE strains [15]. In addition, the highest number of substitutions between the FB and TPE genomes was located in *tpr* genes (*tprC*, *tprI*, *tprF*), which are known to be variable among *T. pallidum* subspecies. Therefore, genetic differences specific for the FB genome appears to be predominantly localized in the variable genomic regions thus suggesting that the observed differences between the FB genomes and other TPE genomes likely do not result in considerable changes in the host range and pathogenicity. In the FB genome, 117 substitutions resulted (except for one nonsense, one start codon and one stop codon mutation) in 88 (75.2%) nonsynonymous mutations (82 nonconserved, 75.1%). A similar percentage of nonsynonymous mutations were also found in other strain-specific changes in TPE genomes (ranging from 66.1 to 80.5%). In this respect, the FB strain is very similar to other TPE strains.

TPE strains appears to be the most ancient treponemes based on the skeletal changes typical for yaws that were identified in bones dated to 1.6 million years ago [49], [50]. Based on whole genome alignments, the FB genome clearly clustered with other TPE strains. Moreover, TPE strains including Fribourg-Blanc clustered separately from TPA strains. Interestingly, almost the same number of nucleotide changes evolutionary separated the *Treponema paraluiscuniculi* Cuniculi A strain from TPE and TPA strains. Assuming that the TPE strains represent ancestral strains, and since it has been suggested that the Cuniculi A genome evolved by genome decay [29], it is possible that this rabbit pathogen evolved sometime during the early evolution of TPA strains (Figure 1) while the FB strain evolved along a similar path as TPE strains; potentially as a result of the close relatedness of its hosts with humans. However, there are several evolutionary scenarios explaining genetic similarity of the Fribourg-Blanc and TPE strains including i) hypothesis that TPE was acquired by humans from nonhuman primates, ii) hypothesis that the Fribourg-Blanc and TPE exchanged regularly between humans and other primates and even iii) possibility that the Fribourg-Blanc represent adaptation of TPE to nonhuman primates. An additional sequence information from other nonhuman primate isolates will be needed to address this question. Moreover, such studies on treponemes isolated from nonhuman primates could help to clarify if TPA evolved from TPE.

Several molecular genetic studies previously suggested that the Fribourg-Blanc strain was very closely related or identical to *T. pallidum* ssp. *pertenue* [18], [19], [21], [22], [23],[24]. A principal finding of this work was the demonstration that the FB genome has similar genetic characteristics as other TPE strains and that the differences specific to the FB genome are similar to those differentiating other TPE strains and located mainly in variable genomic loci. From the results mentioned above, we can infer that the unclassified simian isolate Fribourg-Blanc belongs to the *Treponema pallidum* ssp. *pertenue*. The FB strain was shown to cause experimental infection in human hosts and TPE strains can infect primates. Although the human and animal diseases may be epidemiologically independent, it is likely that a reservoir for yaws exists among primate populations and/or humans serve as a reservoir for baboon infection, especially in Africa. This could considerably complicate recent efforts to eradicate yaws [51]. However, further sequence data on treponemes isolated from nonhuman primates will reveal if these treponemes show molecular signatures similar to FB or human TPE strains. Nevertheless, knowledge of specific FB genetic changes could be useful to the epidemiological aspect of yaws eradication.

## Supporting Information

Table S1 List of primers used for amplification of TP intervals for Fribourg-Blanc strain (primer coordinates, primer sequence, primer length, and TP interval size according to the Nichols strain, GenBank # AE000520.1).
(XLS)

Table S2 List of specific differences between the Fribourg-Blanc (FB) genome and genomes of 3 strains of *Treponema pallidum* ssp. *pertenue* (TPE; Samoa D, CDC-2 and Gauthier).
(XLS)
